# First record of the littoral family Isotogastruridae (Collembola) in Asia

**DOI:** 10.3897/zookeys.136.1666

**Published:** 2011-10-13

**Authors:** Mikhail B. Potapov, Yun Bu, Yan Gao

**Affiliations:** 1 Moscow State Pedagogical University, Kibalchich str., 6, korp. 5, Moscow, 129278 Russia; 2 nstitute of Plant Physiology and Ecology, Shanghai Institutes for Biological Sciences, Chinese Academy of Sciences, Shanghai, 200032 China

**Keywords:** Collembola, Taxonomy, Hainan, China, Tropical area

## Abstract

The new species *Isotogastrura trichaetosa* **sp. n.** isdescribed from a sand beach of Hainan, South China. It differs from all its congeners by 3+3 axial setae on Abd. IV (vs. 2+2) and by the presence of a pair of tubercles on Abd.VI. The geography of this strictly littoral genus is discussed.

## Introduction

Isotogastruridae Thibaud and Najt 1992 is a small family with well developed prothoracic tergit but without seta, together with many special characters shared with families Isotomidae and Hypogastruridae, and thus have an intermediate position between the orders Poduromorpha and Entomobryomorpha (Thibaud and Najt 1992). It is generally accepted that Isotogastruridae rather belongs to Poduromorpha although its phylogenetic position is still not fully understood (Fjellberg 1995).

The single genus *Isotogastrura* of this family includes seven species recorded in the Caribbean Islands (*Isotogastrura arenicola* Thibaud & Najt, 1992), the Canary Islands and Mediterranean (*Isotogastrura coronata* Fjellberg, 1995; Thibaud and Peja 1996), New Caledonia (*Isotogastrura litoralis* Thibaud & Weiner, 1997), Mexico (*Isotogastrura ahuizotli* Palacios-Vargas & Thibaud, 1998, *Isotogastrura veracruzana* Palacios-Vargas & Thibaud, 1998, *Isotogastrura atuberculata* Palacios-Vargas & Thibaud, 2001), and Madagascar (*Isotogastrura madagascariensis* Thibaud, 2008). *Isotogastrura coronata* was also found in Morocco later (Thibaud and Boumezzough 2006). So far all species have been described from littoral sands of tropical areas (Fig. 10).

In the present paper we describe a new species of Isotogasturidaewhich was found in sands of Hainan Island (South China) during a joint project between China and Russia investigating the littoral Collembola of the Pacific coast of Asia. So far it is the first record of the family in Asia.

All specimens were mounted on the slide using Hoyer's solution and dried up for three days in an oven at 60°C.

Abbreviations used in the descriptions are: **Th.** thoracic segment; **Abd.** abdominal segment; **Ant.** antennal segment; **Man.** Manubrium; **s** sensillum/a.

## Taxonomy

### Isotogastrura trichaetosa sp. n.

urn:lsid:zoobank.org:act:A1E1B55F-FA71-4E44-8333-8B94C638A617

http://species-id.net/wiki/Isotogastrura_trichaetosa

#### Material.

Holotype: Female, South China, Hainan Province (western coast), Changjiang County, vicinity of Changhua town, Qizi Bay, 19°21'12"N, 108°40'25"E, beach, flotation of sand samples (No. 34, 35 and 38). 7. IV. 2011, Y. Bu, C.W. Huang, M.B. Potapov and N. A. Kuznetsova leg. Paratype: Three females, same as holotype. Holotype and two paratypes are deposited in Shanghai Institute of Plant Physiology and Ecology, Shanghai Institutes for Biological Sciences, CAS (China); one paratype is deposited at Moscow State Pedagogical University (Russia).

#### Description.

**Figures 1–6.**
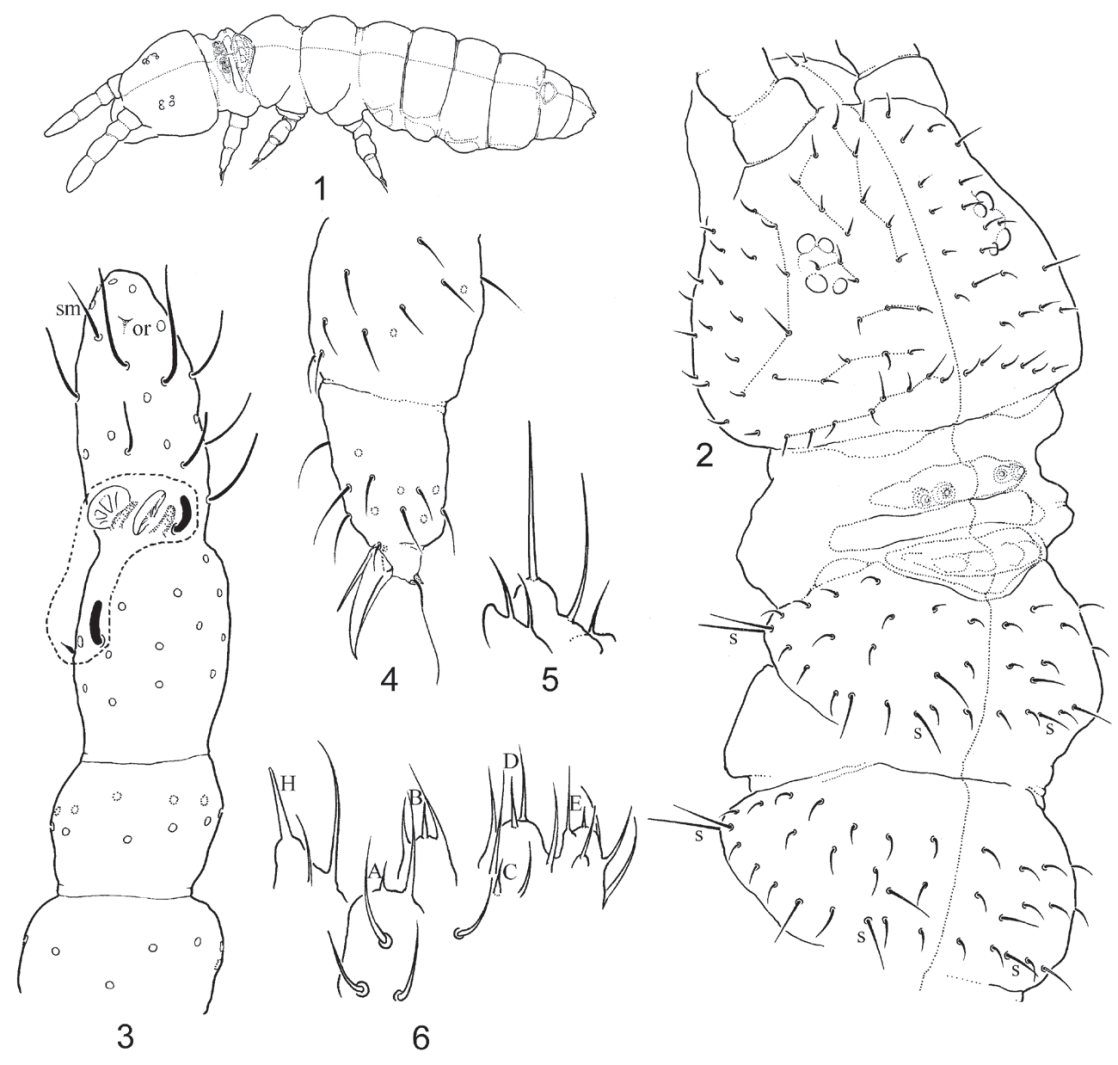
*Isotogastrura trichaetosa* sp. n. **1** habitus, **2** head and thorax, **3** antenna (with antennal organ marked), **4** apical part of Leg 2, **5** maxillary outer lobe (apical palp, sublobal hairs and basal seta shown), **6** labial palp. s - sensillum, or - organite, sm - subapical microsensillum.

**Figures 7–8.**
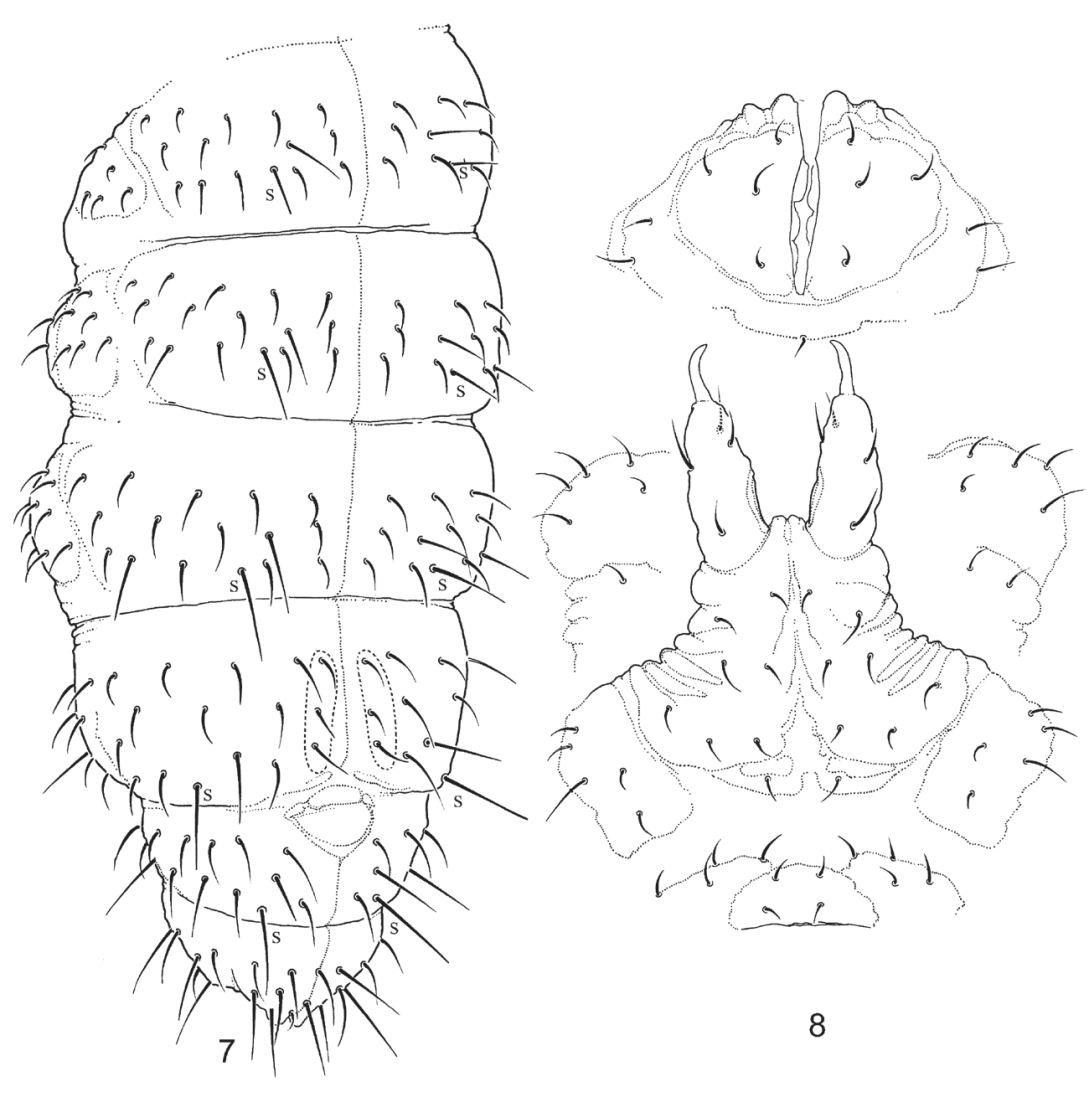
*Isotogastrura trichaetosa* sp. n. **8** dorsal chaetotaxy of abdomen, **9** ventral tube and furcal area (retinaculum not shown).

Body length under slide (n=4): 0.42 mm (range 0.4-0.5 mm), holotype length 0.4 mm. Pale in alcohol, with grey pigmentation uniformly distributed over dorsal areas except for the darker eye patches. Body shape typical of genus, not slender (Fig. 1), without secondary granulation, primary granulation well visible. Head large, with exserted mouth parts as common for the genus (Fig. 2). Ventral side of abdomen wrinkled, especially on manubrium (Fig. 8), which may less visible if the animals are more swollen. Th. I with four dorsal tubercles (Fig. 2). Anterior edge of Abd. V with dorsal glandular opening partly covered by cuticular fold (Fig. 7). One pair of tubercles present at posterior edge of Abd. VI (Fig. 7).

Ant. I and II with 7 (rarely 6) and 11 (rarely 12) setae, respectively. Antennal organ of Ant. III with two granulated cuticular papillae, two blade-like inner sensilla, and two outer tubular simple sensilla, one of which is grouped together with inner ones, the other one positioned more proximally and associated with lateral sensillum, which is small and pointed (Fig. 3). Ant. IV with several thin sensilla, two of which are longer. Subapical organite small and strongly depressed. Subapical microsensillum absent or, less probably, shaped as other setae of the segment (Fig. 3). Labrum with 10 setae grouped together at distal edge as in other species of the genus. Two prelabral minute setae (Fig. 2). Maxillary outer lobe with bifurcate (simple in one individual) apical palp and two sublobal hairs. Branch of apical palp well detached from the main part (Fig. 5). Labium with 5 basolateral and 4 basomedian setae. 3(2)+3(2) postlabial setae, posterior pair of setae absent or positioned more laterally. Hypostomal lobe of labial palp well developed, with strong and thick seta H (Fig. 6). Some elements of labial palp difficult to interpret: apical palps of all papillae (A, B, C, D, E) present but reduced and never beyond (normally shorter) associated guards, papilla E smallest. At least 4 proximal setae and 13 guards (possible variation was not studied because limited number of specimens) (Fig. 6). Mandibles slender as typical for the genus. Maxillary head with most lamellae strong and serrated. Head with 4+4 ocelli, two inner smaller. Postantennal organ absent.

Dorsal chaetotaxy shown is in Figs 2, 7, 8. Th. II-Abd. IV with 3+3 axial setae each. Number of sensilla 2, 2/1,1,1,1,1, microsensilla absent. Sensilla long, with blunt tips, which distinguished from macrosetae. The leg chaetotaxy of subcoxa 1, subcoxa 2, coxa, trochanter, femur, and tibiotarsus is 1,1,4, 6,11,12; 1,3,7, 6,11,12 and 2,3,8-9, 5,10,11 from I to III. Claw and empodium as in Fig. 4, empodium filiform, longer than claw. Thorax without ventral setae. Ventral tube with 6+6 lateral paired setae (4+4 in distal and 2+2 in basal position) and one unpaired posterior seta (Fig. 8). Retinaculum with 3+3 teeth, seta absent. Dens with 1 anterio-median and 3 posterior setae. Manubrium without anterior setae. Posterior side of manubrium with 8+8 setae; subcoxae furcalis with 5+5 setae, (Anterior furcal subcoxa with 5(6), posterior one with 2(1) setae) (Fig. 8). Only females known from the material studied.

#### Remarks.

The new species differs from all congeners by 3+3 axial setae on Abd. IV (vs. 2+2) and by presence of a pair of tubercles on Abd.VI (absent in other species.). *Isotogastrura trichaetosa* sp.n. is the most primitive species of the genus which having more homonomic axial chaetotaxy of abdomen (3,3,3,3) than as common in the genus (3,3,3,2), normal shape of body, and thin sensilla on Ant. IV. Other primitive character, simple (vs. bifurcate) tubular outer sensilla of antennal organ, is shared with *Isotogastrura coronata* Fjellberg, 1995 (Canary Islands) and *Isotogastrura madagascariensis* Thibaud, 2008 (Madagascar).

#### Name derivation.

The new species has 3+3 axial setae on Abd. IV (three setae/chaetae).

#### Distribution and ecology.

**Figure 9.**
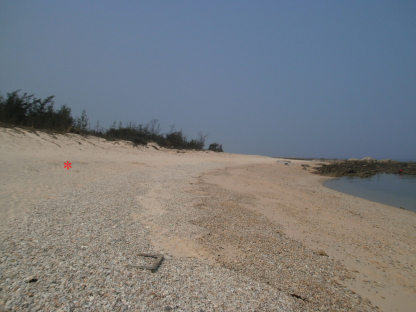
Type locality in Qizi Bay (Southeast China), ★ indicates the sample site.

 The species is known only from the type locality. Small body size of *Isotogastrura trichaetosa* indicates inhabiting narrow passages among the grains of sand. The habitat of other congeners is a fine sand of the upper-littoral zone and thus the genus is ecologically psammobiotic (Thibaud 2007). After the literature data, only *Isotogastrura coronata* penetrates to higher area of littoral, in coastal sand of dunes with roots of halophytes. The type locality of *Isotogastrura trichaetosa* sp. n. is an open coastal beach with very fine sand and some small pebbles (the species was only found in pure sand) and was not recorded by us in the zone of halophytes. Sampling site is shown in Fig. 9.

#### The geography of the genus.

**Figure 10.**
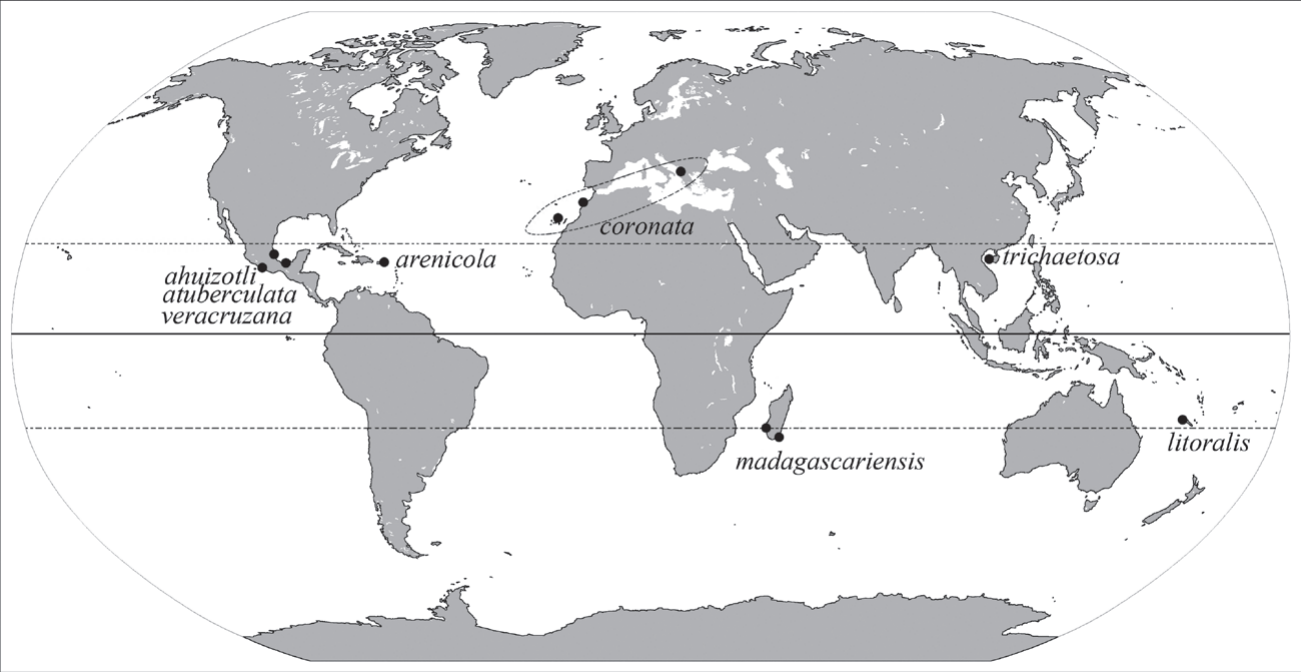
The records of the species of genus *Isotogastrura*

 The most species of the genus occur between the Tropic of Capricorn and the Tropic of Cancer, except *Isotogastrura coronata* penetrating to Mediterranean (Fig. 10). Our record indicates that *Isotogastrura* is also distributed in the tropical Asia and thus make the genus completely pantropical. Usually, littoral species are distributed widely along the coasts due to transport possibilities by water and similar conditions of the habitat. In Collembola, Thibaud (2007) remarked many species from interstitial littoral sands having all a trans-oceanic distribution. High ability of water dispersal was not experimentally confirmed for these species but so was done by Coulson et al. (2002) in five species of Collembola distributed in Arctic. In other groups, littoral species distributed widely along the sea coast are also well known (Chernov 1997), for instance seaweed and beach flies *Coelopa frigida* (Fabricius, 1805) and *Fucellia maritima* (Haliday, 1838). Contrary to this trend the genus *Isotogastrura* so far shows the considerable geographical segregation of locally distributed species.

## Supplementary Material

XML Treatment for Isotogastrura trichaetosa
